# Reply to: Ventriculitis incidence and outcomes in patients with aneurysmal subarachnoid hemorrhage: a prospective observational study

**DOI:** 10.62675/2965-2774.20260095

**Published:** 2026-07-17

**Authors:** Ricardo Turon, Pedro Kurtz, Carla Rynkowski, Letícia Petterson, Bruno Gonçalves, Vanessa de Caro, Marco Prazeres, Fernando Augusto Bozza, Cássia Righy

**Affiliations:** 1 Instituto Estadual do Cérebro Paulo Niemeyer Department of Neurointensive Care Rio de Janeiro RJ Brazil Department of Neurointensive Care, Instituto Estadual do Cérebro Paulo Niemeyer - Rio de Janeiro (RJ), Brazil.; 2 Hospital Cristo Redentor Department of Intensive Care Medicine Porto Alegre RS Brazil Department of Intensive Care Medicine, Hospital Cristo Redentor - Porto Alegre (RS), Brazil.; 3 Instituto D’Or de Pesquisa e Ensino Rio de Janeiro RJ Brazil Instituto D’Or de Pesquisa e Ensino - Rio de Janeiro (RJ), Brazil.

## TO THE EDITOR

We sincerely thank Dr. Josef Finsterer, Dr. Carla Alessandra Scorza, and Dr. Fulvio Alexandre Scorza,^(1)^ as well as Dr. Julya Santana Alves de Barroso, Dr. Henri Dourado de Almeida, Dr. Amanda Cirilo de Oliveira Lins, Dr. Jorge Fernando Pereira Silva, Dr. Achilles de Souza Andrade, and Dr. Johnnatas Mikael Lopes,^(2)^ for their interest in our study and for their thoughtful methodological and statistical comments. We appreciate the opportunity to clarify important aspects of our study design and analytical approach related to ventriculitis incidence and outcomes in patients with aneurysmal subarachnoid hemorrhage (SAH).^(3)^

### Study aim and clinical criteria

In their letter, Finsterer et al. raised concerns regarding the lack of specification of risk factors for external ventricular drain (EVD) placement and subsequent infection, suggesting that variables such as age, gender, comorbidities, concomitant medications, severity of bleeding, blood volume, ventricular intrusion, and intraventricular thrombolysis should be explored in this context.^(1)^ The primary aim of our study, however, was to evaluate the incidence of ventriculostomy-associated infection (VAI) and its impact on clinical outcomes, rather than to identify risk factors for EVD placement or for ventriculitis.^(1,3)^ As shown in tables 1 and 2 of our article, 89% of patients presented modified Fisher grades 2 - 4 and 66% had hydrocephalus at admission, justifying the indication for EVD.^(3)^ The decision to insert the drain was based on standardized clinical and radiological criteria (acute hydrocephalus, decreased consciousness due to hydrocephalus, or need for intracranial pressure monitoring), minimizing indication bias.^(1,3)^ No patient received intraventricular thrombolysis, as also questioned by Finsterer et al.^(1)^

### Impact of the COVID-19 pandemic, delayed cerebral ischemia diagnosis, and clarification on temporal analyses

Finsterer et al. also questioned the potential impact of severe acute respiratory syndrome coronavirus 2 (SARS-CoV-2) infection (SC2I) on the spectrum of infectious agents and outcomes in our cohort, given that the study period spanned from July 2015 to December 2020, encompassing the early stages of the pandemic.^(1)^ During the pandemic, all patients were systematically tested for SARS-CoV-2 prior to intensive care unit (ICU) admission, and only negative cases were included in the study.^(1,3)^ Therefore, there was no coexistence of infected and noninfected patients within the cohort, and no modifications were made to institutional diagnostic or therapeutic protocols during the study period.^(1,3)^

In the same letter, the authors queried how delayed cerebral ischemia (DCI) was diagnosed and distinguished from other causes of neurological deterioration, particularly in the context of ischemic stroke as a complication of SC2I.^(1)^ In our study, the diagnosis of DCI was primarily clinical and based on established neurocritical care recommendations, with imaging confirmation when applicable, after systematic exclusion of alternative causes of neurological worsening such as rebleeding, infection, or intracerebral hemorrhage.^(1,3)^ This clarification addresses the concerns raised by Finsterer et al. regarding the diagnostic accuracy of DCI.^(1)^

About the comment raised by Finsterer et al., suggesting that the latency between ictus and EVD placement, as well as the latency between aneurysm treatment (coiling, clipping, or no treatment) and EVD insertion, should be further analyzed,^(1)^ we respectfully emphasize that this was not the objective of the present study. Our investigation was specifically designed to evaluate the incidence of VAI and its association with mortality and functional outcomes.^(1,3)^ The timing of EVD placement as a determinant of infection risk, as explored in other contexts such as shunt dependency,^(4)^ was beyond the predefined scope of our study.^(1)^

### Additional methodological aspects

Finsterer et al. also pointed to an apparent inconsistency between including "all consecutive" patients in the methods section and excluding those admitted more than 30 days after ictus, pregnant women, and those with a life expectancy < 48 hours.^(1)^ We acknowledge this discrepancy in wording and clarify that all consecutive patients meeting the inclusion criteria were enrolled.^(1,3)^ Both participating hospitals are tertiary referral centers for aneurysmal SAH within the Brazilian public health system, and diagnosis is generally established before transfer.^(1,3)^ Some patients are referred more than 30 days after the initial ictus due to the structure of the regional referral system.^(1,3)^ According to our predefined exclusion criteria, patients admitted more than 30 days after hemorrhagic onset were excluded to maintain homogeneity regarding acute-phase complications.^(1,3)^ However, all included patients underwent diagnostic cerebral angiography, confirming aneurysmal etiology.^(3)^

Regarding the concern raised by Finsterer et al. about patients diagnosed with SAH by xanthochromia detection on cerebrospinal fluid examination when cerebral computed tomography was negative,^(1)^ we clarify that xanthochromia testing, when required, was performed at the referring institutions but is not routinely conducted at our centers once aneurysm diagnosis has been established, in line with previous recommendations on thunderclap headache workup.^(5)^ All included patients had confirmed aneurysmal SAH by either initial computed tomography scan or by xanthochromia in cerebrospinal fluid, followed by cerebral angiography.^(1,3,5)^

Regarding moribund patients and those with expected survival < 48 hours, Finsterer et al. questioned how it could be determined that patients would die within 48 hours and why pregnant women were excluded.^(1)^ This was a clinical determination made at ICU admission, based on catastrophic neurological injury (such as clinical signs compatible with brain death) and multidisciplinary consensus.^(1,3)^ Pregnant women were excluded due to the potential confounding effects of pregnancy-related physiological changes on infection risk, inflammatory markers, and outcomes, as well as ethical considerations regarding radiation exposure during diagnostic imaging and aneurysm treatment procedures.^(1,3)^

### Temporal data and outcomes

Both letters highlighted the importance of temporal variables, including the interval between ictus and EVD placement and between EVD placement and infection.^(1,2)^ Our study presents these key temporal data: the time from ictus to EVD placement was 3 days (interquartile range [IQR] 1 - 7), and from EVD placement to VAI diagnosis was 6 days (IQR 4 - 9).^(3)^ These intervals reflect the clinical latency relevant to infection risk, aligned with the study's focus on incidence and outcomes rather than comparison between EVD and non-EVD groups.^(1-3)^ As previously stated, our objective was not to compare EVD *versus* non-EVD groups or to analyze predictors of EVD placement, but rather to evaluate whether VAI influenced mortality or functional outcomes.^(1-3)^

### Follow-up and functional assessment

Finsterer et al. expressed concerns about the use of telephone interviews for 12-month outcome assessment, noting the potential disadvantages that the accuracy of information cannot be easily verified, that it is not certain the patient himself is answering the questionnaire rather than a relative or caregiver, and that the calculated modified Rankin Scale (mRS) may not be accurate.^(1)^ In our study, 12-month outcomes were assessed through structured telephone interviews using the validated Brazilian Portuguese version of the modified Rankin Scale, which showed excellent agreement with in-person evaluation (weighted κ = 0.89).^(7)^ This method ensured reliable outcome assessment and minimized loss to follow-up given the geographic and socioeconomic context of our multicenter cohort.^(3,7)^ We acknowledge the generic limitations of telephone surveys discussed in public health research,^(6)^ but emphasize that the use of a structured, validated instrument specifically developed for Brazilian stroke patients mitigates many of these drawbacks.^(7)^

Patients presenting at admission with clinical signs compatible with brain death or moribund status were excluded, as previously noted and as queried by Finsterer et al.^(1,3)^ All outcome assessments were based on structured clinical evaluation protocols validated for the Brazilian population.^(3,7)^

### Statistical approach and justification

Barroso et al. questioned the use of logistic regression and suggested that Cox regression would be more appropriate for our prospective study, also discussing issues related to statistical power and loss to follow-up in our cohort.^(2)^ They correctly noted that using the odds ratio (OR) to estimate effect in prospective studies can produce oversized interval measures and suggested that Cox regression with hazard ratio (HR) would be preferable, particularly given the loss to follow-up exceeding 10%.^(2,8,9)^

We appreciate this important methodological discussion. In our study, outcomes were binary and measured at fixed time points (hospital discharge and 12 months); thus, logistic regression was the most appropriate model to estimate associations, adjusting for key prognostic variables (age, World Federation of Neurological Surgeons scale [WFNS], modified Fisher scale, global cerebral edema, and pneumonia).^(2,3)^ The Cox model is primarily indicated for time-to-event analyses and assumes proportional hazards throughout the follow-up period,^(2)^ which was not the focus of our research question. Our study was not designed or powered to detect differences in hazard over time or to analyze survival curves, but rather to compare outcome status (favorable versus unfavorable functional outcome and mortality) at predefined endpoints.^(2,3,8)^

We agree with Barroso et al. that loss to follow-up can affect risk estimation in longitudinal studies and that effect measures such as odds ratios may overestimate risk ratios under certain conditions, particularly in prospective cohorts where the outcome is common.^(2,9)^ However, in our cohort, sensitivity analyses comparing baseline characteristics (age, WFNS grade, modified Fisher scale, comorbidities, and complications) of patients with and without 12-month follow-up did not reveal significant differences (see Supplementary Tables 2S to 5S in the original manuscript),^(3)^ supporting the internal validity of our main findings despite a 26% loss to follow-up.^(2,3)^ This suggests that loss to follow-up was likely random rather than systematically related to exposure or outcome status.^(2,3)^

Regarding the statistical power calculations presented by Barroso et al.,^(2)^ we acknowledge that our sample size (271 patients with EVD, 127 with VAI) was designed to detect clinically meaningful differences in the primary outcomes (mortality and poor functional outcome defined as mRS 4 - 6), but was not powered to detect small effect sizes (10% difference in HR) in survival analyses. The lack of statistically significant association between VAI and outcomes in our study may reflect either a true null effect or insufficient power to detect small differences, as appropriately highlighted by Barroso et al.^(2)^

## CONCLUSION

We appreciate the opportunity to clarify these important methodological points and to engage in a constructive dialogue with Barroso et al.^(2)^ and Finsterer et al.^(1)^ The comments raised highlight the relevance of methodological transparency and alignment between analytical models and study objectives.^(1,2,8,9)^ In the context of our research question – to determine whether VAI was associated with worse outcomes at fixed time points – logistic regression adequately addressed the objective, and the study design ensured clinical and statistical consistency.^(2,3,8,9)^

Future studies with larger sample sizes and longer follow-up may benefit from survival analyses using Cox regression to examine time-to-event outcomes and potential time-varying effects of VAI,^(2,8)^ as well as from more detailed exploration of risk factors for EVD-associated infections and their microbiological profiles across different healthcare settings.^(1,3)^
